# Competitive Ability Effects of *Datura stramonium* L. and *Xanthium strumarium* L. on the Development of Maize (*Zea mays*) Seeds

**DOI:** 10.3390/plants10091922

**Published:** 2021-09-15

**Authors:** Hassan Karimmojeni, Hamid Rahimian, Hassan Alizadeh, Ali Reza Yousefi, Jose L. Gonzalez-Andujar, Eileen Mac Sweeney, Andrea Mastinu

**Affiliations:** 1 Department of Agronomy and Plant Breeding, College of Agriculture, Isfahan University of Technology, Isfahan 84156-83111, Iran; kmojeni@cc.iut.ac.ir; 2 Department of Agronomy and Plant Breeding, University of Tehran, Karaj 77871-31587, Iran; hrahimian@ut.ac.ir (H.R.); malizade@ut.ac.ir (H.A.); 3 Department of Plant Production & Genetics, University of Zanjan, Zanjan 45195-313, Iran; 4 Instituto de Agricultura Sostenible (CSIC), 14004 Cordoba, Spain; andujar@ias.csic.es; 5 Department of Molecular and Translational Medicine, University of Brescia, 25123 Brescia, Italy; e.macsweeney@studenti.unibs.it

**Keywords:** common cocklebur, jimsonweed, geometric characteristics, weed competition

## Abstract

The objective of this study was to explore the physical properties of maize seeds in competition with weeds. The basic and complex geometric characteristics of seeds from maize plants, competing with *Datura stramonium* L. (DS) or *Xanthium strumarium* (XS) at different weed densities, were studied. It was found that the basic and complex geometric characteristics of maize seeds, such as dimension, aspect ratio, equivalent diameter, sphericity, surface area and volume, were significantly affected by weed competition. The increase in weed density from 0 to 8 plants m^2^ resulted in an increase in the angle of repose from 27° to 29°, while increasing weed density from 8 to 16 plants m^2^ caused a diminution of the angle of repose down to 28°. Increasing the density of XS and DS to 16 plants m^2^ caused a reduction in the maximum 1000 seed weight of maize by 40.3% and 37.4%, respectively. These weed side effects must be considered in the design of industrial equipment for seed cleaning, grading and separation. To our knowledge, this is the first study to consider the effects of weed competition on maize traits, which are important in industrial processing such as seed aeration, sifting and drying.

## 1. Introduction

The harmful effects of weeds include crop growth inhibition, added protection costs, reduced farm products quality, reduced animal products quality, increased production costs, reduced harvest efficiency and increased processing costs, with resulting effects on water management and human health [1,2,3,4,5,6,7]. There are many negative aspects of weeds but one of the most important is that of their negative effects on the physical and engineering properties of seeds, an area that there has been very little study.

Weeds change the physical and engineering properties, geometric diameter, sphericity and the seed three axial dimensions, bulk density, length, width and thickness, coefficient of friction and aspect ratio, of crop seeds and can cause reductions in machinery efficiency and product losses. Many problems with the design of agricultural machinery are associated with the physical and engineering properties of crops, and the analysis of product behaviour during agricultural processing operations such as handling, planting, harvesting, threshing, cleaning, sorting and drying is fundamental to resolving this problem [8,9,10,11,12]. These properties are important in the construction of bulk storage facilities and the calculation of intermediate holding bin dimension capacity. Problems associated with bin design should not be attributed to disagreement among design philosophies, but rather to a serious lack of understanding of certain grain properties and how they relate to bin design [13,14,15].

Principal axial dimensions of maize seeds are useful in selecting sieve separators and in calculating the necessary power during the maize milling process. They can also be used to calculate surface area and kernels volume, which are important during modelling of, seed drying, aeration and heating. Maize seed flow ability is usually measured using the angle of repose (a measure of the internal friction between kernels) that is useful in hopper design, since the hopper wall’s inclination angle should be greater than the angle of repose to ensure the continuous flow of the materials by gravity [11].

The size of the individual seed also plays a major role in the seed germination quality. Large sized seeds in pigeon pea recorded a higher percentage of germination and field emergence [16,17,18]. These properties are affected by numerous factors such as size, form, moisture content of the grain [19] and varieties [20]. Sandhu and colleagues showed that the 1000 kernel weight and bulk density varied significantly according to maize varieties [21]. Many studies have been reported on the physical properties of fruits, grains and seeds, such as rough rice (*Oryza sativa* L.) [11,22,23], cowpea seed (*Vigna sinensis* L.) [24], lentil (*Lens culinaris* Medik) [25], maize [21], safflower (*Carthamus tinctorius* L.), [26], aonla (*Emblica officinalis* Gaertn) [27], nutmeg seeds (*Monodora myristica*) [28], chickpea seeds [29] or others [4,10,15,30,31,32,33,34], but no detailed study concerning the effect of weeds on the physical and engineering properties of maize seed has been carried out.

Jimsonweed (*Datura stramonium* L.) and common cocklebur (*Xanthium strumarium* L.) are thought to be the most competitive weeds to be found in maize fields [35]. *Datura stramonium* is a troublesome weed to be found in almost 100 countries, interfering with the production of more than 40 crops. It is one of the 10 most costly weeds to crop production plant in the eastern states (e.g., North Carolina and Virginia) of the United States [36]. *Xanthium strumarium* is an adaptable species distributed worldwide [37], with a central or South America origin but is now widespread throughout North America, Europe, Asia, parts of Australia and Africa [38]. It is one of the 10 most common cotton crop weeds in some states of the U.S. [39].

In the crop–weed mixture, the crop plant susceptibility varies depending on the weed density, duration of competition and competitive ability of weed species [40,41]. In agroecosystems, dormancy, germination and emergence of weed seeds are under the influence of the environmental factors such as temperature, light, nitrate, seed burial depth and moisture [42]. Travlos and colleagues showed that the effect of these factors varies between species. Therefore, crop fields differ considerably in the number of emerging species (weed spectrum) and number of individuals of the same weed species (weed density) [42].

Weed growth alongside the crop plants can limit the resources such as nutrients, moisture, light and space that are necessary for proper growth, resulting in reduced crop growth, and ultimately, the size, shape, colour and weight of individual seed will be affected by competition [43]. The weed effects on maize growth and productivity in term of seed yield, biomass, etc., previously have been well studied; however, to the best of knowledge, properties that affect post-harvest operation have not been studied. Therefore, the objective of this study was to explore the effect of weeds (e.g., *D. stramonium* and *X. strumarium*) on the physical properties of maize seed and the resulting effect on the design of handling, processing and packaging machinery for maize production.

## 2. Materials and Methods

### 2.1. Experimental Site and Maize Seed Producing Procedures

The experiment was conducted between 2006 and 2007 in the Research Field of Paradise of Agriculture and Natural Resources, University of Tehran, Karaj (35°34′ latitude N, 50°57′ longitudes E and 1160 m a.s.l) Iran. The soil of the test site for both years was clay loam (28.4% sand, 35% silt and 33.6% clay, pH 5.7 and 0.61% organic matter in 2006 and 1.67% organic matter in 2007). Seedbed preparation, for both years, consisted of mouldboard ploughing (20–25 cm) and tandem disking followed by land leveller smoothing in the spring prior to planting. Plots were fertilized with 250 kg ha^−1^ of ammonium phosphate and 150 kg ha^−1^ of urea in the spring before planting. Additional N (200 kg ha^−1^ of urea) was added to plots through water irrigation with the maize at the 6–8 leaf stage. Clorpirifos (Golsam Gorgan Chemicas Co.; 1.5 L ha^−1^) was applied twice in the early season to protect both the maize and the weeds against *Agrotis* species.

The plot was 3 m wide by 10 m long with four rows spaced 75 cm apart. Maize (cv. single cross hybrid 704) was seeded at a plant density of 6.06 plants m^2^ on 5 May 2006 and 2007. *X. strumarium* and *D. stramonium* were hand planted the same date on both sides of the maize rows 15 cm distant from the crop.

**Figure 1:**
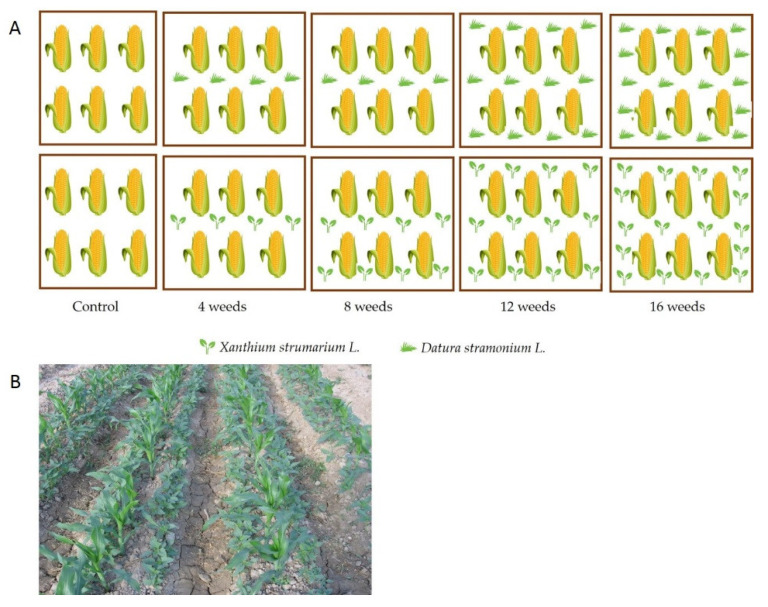
(**A**) Graphical scheme of study design. Maize was seeded at a plant density of 6.06 plants m^2^ and weed species (*D. stramonium* and *X. strumarium*) at a plant density of 4, 8, 12 and 16 plants m^2^. (**B**) Maize and weeds crops from Research Field of Paradise of Agriculture and Natural Resources, University of Tehran, Karaj.

The experiment was conducted according to a randomized complete block factorial design with three replications. The variables studied included weed density (four levels: 4, 8, 12 and 16 plants m^2^) and weed species (*D. stramonium* and *X. strumarium*). Weed-free plots (weed density = 0) was also included as control. Maize and weed seedlings were thinned to the target densities at their 2–4 leaf stages. Plots were maintained free of weeds other than *X. strumarium* or *D. stramonium* throughout the growing season by hand weeding (Figure 1).

Maize seeds were harvested from the two central rows in an area measuring three m^2^. These seeds were oven dried for 72 h at 72 °C to bring the seed moisture to the standard level and were then stored in cloth bags in a refrigerator (5 ± 2 °C) until their physical properties were measured.

### 2.2. Physical Properties Measurement

The maize seeds (cv. Sc 704) obtained in 2006 and 2007 (as described in the Experimental Site and Procedures section) were used for all the experiments in this study. The seeds were manually cleaned and all foreign matter, such as straw and dirt, was removed. The seed moisture content used in the experiments ranged from 14.3 to 15.4% dry base.

### 2.3. Size and Sphericity

Thirty seeds were selected from each plot (seeds from the middle of the ears and seeds from the top and the bottom of ears were not considered because their shape and form were too irregular). The seeds linear dimensions, namely, length (*L*), width (*W*) and thickness (*T*), were measured using a digital Vernier calliper with a precision of 0.01 mm. The sphericity of the maize seeds were calculated using the following formula [44]:

(1) $$\varphi =\frac{{\left(LWT\right)}^{\frac{1}{3}}}{L}$$

where φ is the sphericity; *L* is the length in mm; *W* is the width in mm; *T* is the thickness in mm.

### 2.4. Equivalent Diameter

The equivalent diameter (*D_p_*) in mm considering a prolate spheroid shape for maize seed was calculated using the following formula [44]:

(2) $$D_{P}={\left(L\frac{{\left(W+T\right)}^{2}}{4}\right)}^{\frac{1}{3}}$$

### 2.5. Seed Volume and Surface Area

Seed volume (*V*) and surface area (*S*) were calculated using the following formulae:

(3) $$V=0.25\left[\left(\frac{\pi }{6}\right)L{\left(W+T\right)}^{2}\right]$$

(4) $$S=\frac{\pi BL^{2}}{\left(2L-B\right)}$$

(5) $$\mathrm{Where},\;B=\sqrt{WT}$$

### 2.6. Aspect Ratio

The aspect ratio (*R_a_*) of the seed shape was calculated as:

(6) $$R_{a}=\frac{W}{L}$$

### 2.7. Seeds Weight

In order to determine the one thousand seeds weight, an electronic counting machine (Numigral, ELE International Limited, Leighton Buzzard, UK) counted one hundred maize seeds, these seeds were weighted by means of an electronic balance with a precision of 0.01 g, and the resulting weight was extrapolated to the weight for 1000 seeds.

### 2.8. Angle of Repose

The angle of repose is the angle with the horizontal at which the material will stand when piled. This was determined by using an apparatus consisting of a plywood box (140 × 160 × 35 mm^3^) with two fixed and adjustable plates. The box was filled with the sample, and then, the adjustable plate was inclined gradually allowing the seeds to assume a natural slope, which was measured as the emptying angle of repose [11].

### 2.9. Statistical Analysis

The data were analysed using ANOVA with the PROC GLM procedure of SAS version 9.1 (SAS Institute, 2002, Cary, NC, USA). Before analysing the data, the assumption of a homogeneous variance was tested. If the assumption was not adequately met, data were subjected to invers (1/x) transformation. If the analysis of variance indicated statistically significant differences, the means were compared using Tukey’s studentized range test (*p* > 0.05).

## 3. Results

A combined analysis of 2006 and 2007 experiments showed a non-significant effect of the year × treatment (weed species and density) interaction on maize seed properties (Table 1). Therefore, data from two experiments were pooled.

### 3.1. Basic Geometric Characteristics

The analyses of variance of the effects of weed species, density and their interaction on the basic geometric characteristics of the maize seeds in terms of length, width, thickness, aspect ratio and equivalent diameter are illustrated in Table 1. It was observed that characteristics such as dimension, aspect ratio and equivalent diameter were significantly affected by weed density (*p* ≤ 0.01). However, the basic geometric characteristics were not affected by the weed species and *X. strumarium* and *D. stramonium* had the same effect on these characteristics.

Based on combined analysis (Table 1), the seed length diameter showed higher mean values for 2007 (12.08 mm) when compared to those for 2006 (11.55 mm). However, other characteristics such as width and thickness did not vary according to the year. The maize seeds in weed-free plots had the highest values for all the geometrical characteristics (other than that for aspect ratio) and an increasing weed density caused a decrease in these values (Table 2). Aspect ratio, on the other hand, generally increased with increasing weed density and reached 68.16 at a density of 16 plants m^2^.

### 3.2. Complex Geometrical Characteristics

**Figure 2:**
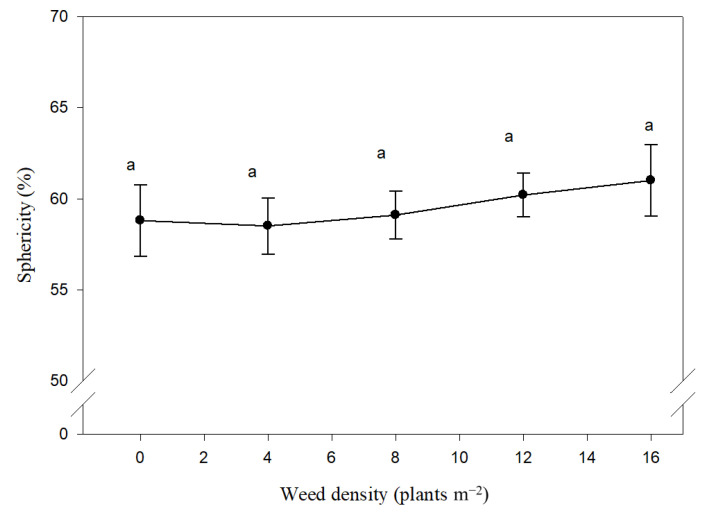
Effect of different density of *Xanthium strumarium* and *Datura stramonium* on maize seed sphericity. Mean values with the same letters are not significantly different based on Tukey’s studentized range test (*p* ≤ 0.05). Vertical lines are standard error of mean (*n* = 12; two years, two weed species and three replications).

**Figure 3:**
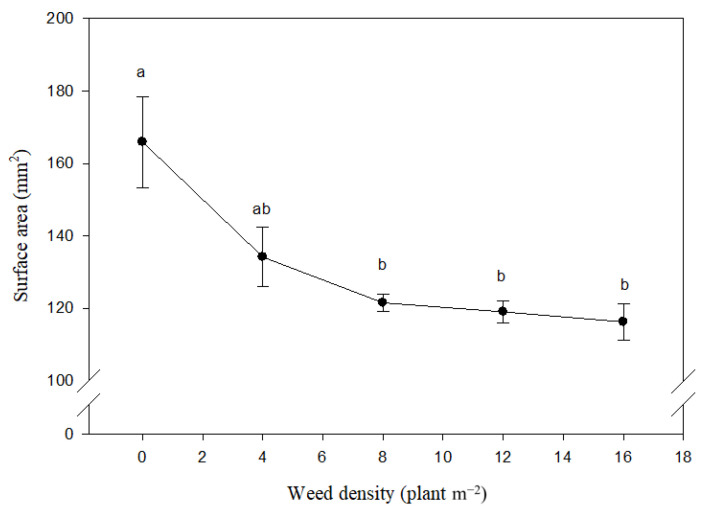
Effect of different density of *Xanthium strumarium* and *Datura stramonium* on maize seed surface area. Mean values with the same letters are not significantly different based on Tukey’s studentized range test (*p* ≤ 0.05). Vertical lines are standard error of mean (*n* = 12; two years, two weed species and three replications).

**Figure 4:**
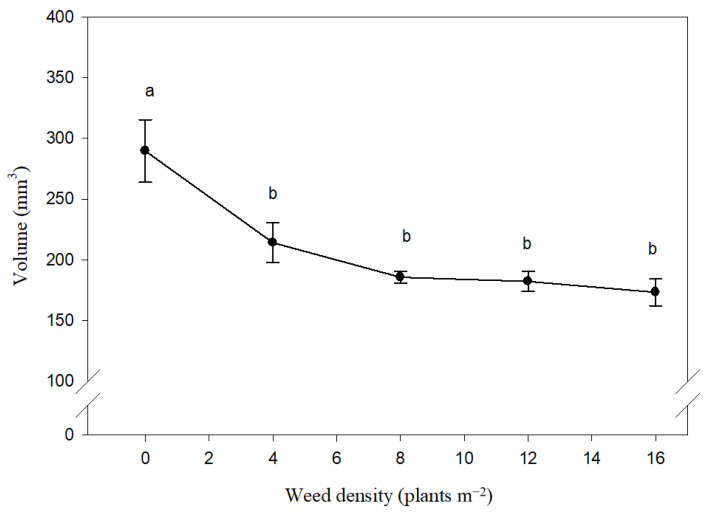
Effect of different density of *Xanthium strumarium* and *Datura stramonium* on maize seed volume. Mean values with the same letters are not significantly different based on Tukey’s studentized range test (*p* ≤ 0.05). Vertical lines are standard error of mean (*n* = 12; two years, two weed species and three replications).

The complex geometrical characteristics such as sphericity, surface area and volume are illustrated in Figure 2, Figure 3 and Figure 4, respectively. Based on combined analysis, sphericity is not affected by weed species, density and their interaction. In weed-free conditions, on average, over the two years studied, the maize seed sphericity was 58%, while with increasing weed density up to 16 plant m^2^, sphericity increased to 61% (Figure 2).

The maize seed surface area was not affected by weed species, while the weed density had significant effects (Table 1). Averaged over the two-year period, an increase in weed density from 0 to 16 plants m^2^ caused the surface area of the maize seeds to decrease from 165.89 mm^2^ to 116.21 mm^2^ (Figure 3).

Seed volume was also significantly affected by weed density, while weed species and interaction of density × species was not significant (Table 1). The volume of the maize seeds decreased from 289.70 mm^3^ to 173.28 mm^3^ while weed density increased from 0 to 16-plant m^2^ (Figure 4).

### 3.3. Seed Weight

**Figure 5:**
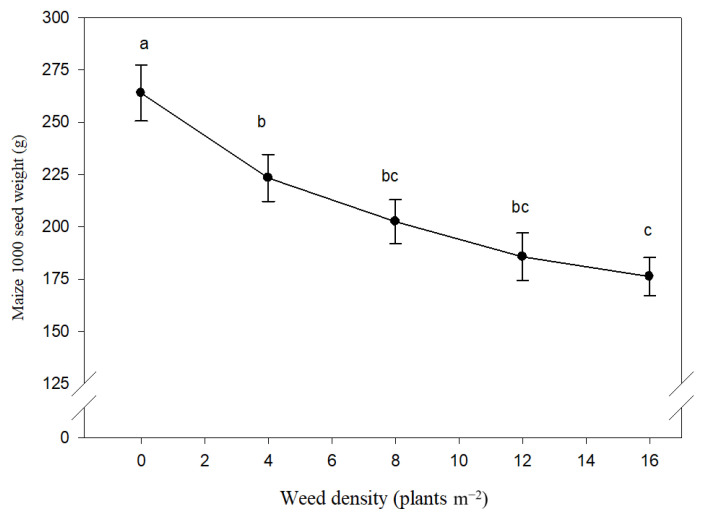
Effect of different density of *Xanthium strumarium* and *Datura stramonium* on maize 1000 seed weight. Mean values with the same letters are not significantly different based on Tukey’s studentized range test (*p* ≤ 0.05). Vertical lines are standard error of mean (*n* = 12; two years, two weed species and three replications).

The one thousand seed weight was significantly affected by weed density, while species and their interaction were not significant (Table 1). Average over weed species and density, seed weight was greater in 2006 (216 g) than 2007 (204 g). Averaged over two years, the decrease in seed weight was linear with the increasing weed density (Figure 5). In 2006, the weed-free 1000 seed weight of maize was 267 g, while it decreased to a value of 175 g for 16 *X. strumarium* plants m^2^ and to a value of 204 g for 16 *D. stramonium* plants m^2^. In 2007, the 1000 seed weight for maize (265 g) diminished by 40.3% when the density of *X. strumarium* was 16 plants m^2^. The same weed density of *D. stramonium* resulted in a reduction of 37.4% of the 1000 seed weight.

### 3.4. Angle of Repose

**Figure 6:**
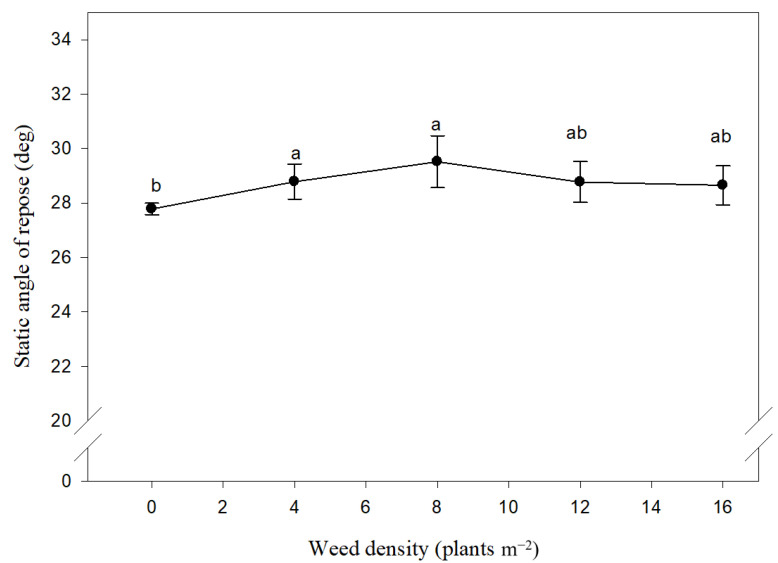
Effect of different density of *Xanthium strumarium* and *Datura stramonium* on angle of repose of maize seeds. Mean values with the same letters are not significantly different based on Tukey’s studentized range test (*p* ≤ 0.05). Vertical lines are standard error of mean (*n* = 12; two years, two weed species and three replications).

The results for the maize seed static angle of repose compared to the weed density (men over the two years studied) are shown in Figure 6. Under weed-free conditions, the static angle of repose of maize seed remains constant over the two years. However, the presence of competitive *X. strumarium* and *D. stramonium* resulted in a significant change in the angle of static repose (Figure 6). On average over the two-year period, an increase in weed density from 0 to 4 plant m^2^ caused an increase in the angle of repose from 27 to 28°, while an increase in weed density from 4 to 16 plant m^2^ had no significant effect on the maize seed angle of repose.

## 4. Discussion

Various types of cleaning, grading and separation equipment are designed based on the physical properties of the seeds. Therefore, determination of the physical properties of seeds is essential for the design of planting, harvesting, handling, conveying, drying, aeration, storing and dehulling equipment [45]. In this study, all dimensions decreased with an increase in weed density from 0 to 16 plant m^2^. It is widely accepted that increasing weed density can lead to a detrimental impact on the crop plants attributes. Our finding showed that physical properties could also be affected by the level of weed competition conditions that the mother plants were grown. Higher weed density impedes photosynthetic activity (both by shading and by depleting nutrients of rhizosphere) in the mother plant causing the production of less photosynthetic products, nutrients and moisture, which ultimately leads to a lower seed weight and size. Weed competition, which occurred in the early development growth stage of maize, delayed the rate of leaf appearance [46]. Similar responses were observed by Steinmause and Norris (2002) [47]. In this work, competition by the weeds reduced the leaf area index (LAI) of maize severely (data not shown). With decreasing LAI, the interception of photosynthetic active radiation will decrease, and ultimately, the rate of photosynthesis and carbohydrate, which is needed for seed development and growth, will decrease [48]. Shape, the degree of its ability to roll, length, surface texture weight and surface characteristics of most of the crop seeds are used for cleaning and grading. In the current work, with an increase in weed densities form 0 to 16, the seed length, width and thickness were found to decrease by 19.6, 11.3 and 19.8%, respectively (Table 2). It is clear that different reductions in seed dimension can change its shape, and as a result, efficacy of sowing, harvesting, transporting, handling and processing equipment, which work based on seed shape, can decrease. Su et al. (2020) found that kernel shape is the detrimental factor affecting the rupture force and energy of maize kernels [49]. They reported that at the breadth position, pyramidal and rectangular shape kernels exhibited the largest force energy and rupture, compared to round kernels.

Weeds interference with maize can modify crop morph physiology [50], dry matter production and plant growth [51] and yield loss [46,51]. Weed species kind, densities and their interactions influence maize performance [51]. *Xanthium strumarium* compete with maize water, nutrients, space, etc., which finally reduce the yield up to 67% when it present at total crop growth stages [51]. While Massinga and colleagues reported that the yield loss in maize could be 91% by competition of some problematic species such as amaranth (*Amaranthus palmeri* S. Wats) [52]. Weed species differ in their competitive ability, and usually, they are species that grow and spread rapidly by capturing more ground cover. Hence, weed species show a competitive advantage over slower growing plants. Consequently, similar densities of two different species may not have similar effects in certain crops [53]. For example, Dhima and colleagues found that *Avena sterilis* at a density of 120 m^2^ was more competitive than 400 *Phalaris minor* m^2^ [54]. Previous studies found that certain agronomic traits of maize could be more affected by *X. strumarium* than *D. stramonium*. The differences were attributed to the height and biomass production ability of *X. strumarium*. The greater biomass production of *X. strumarium* results in a greater demand for resources, reducing the availability for maize [51]. One of the main factors that provides advantage to *X. strumarium* in competition with maize (compared to *D. stramonium*) could be its similar height to maize, especially in the early and mid-growing season. However, in this work, the negative effect of *X. strumarium* on the physical properties of maize seeds was the same as that of *D. stramonium*. This implies that this effect is not species specific, but the mechanisms remain unclear. The absence of interaction was also probably due to the same effects of two species.

Physical and mechanical properties such as bulk density, true density and porosity can also be important in sizing grain hoppers and storage facilities. These properties can affect the rate of heat and moisture mass transfer during aeration and drying processes [11]. Grain beds with low porosity resist more to water vapour escape during the drying process, which leads to the use of more power to drive the aeration fans [11]. Additionally, the static coefficient of friction is used to determine the angle at which chutes must be positioned in order to achieve consistent flow of materials through the chute and also has an important role in transportation (loading and unloading) of goods and in storage facilities [24]. Such information is important in sizing motor requirements for grain transportation and handling. The focus of this work was to examine the alterations on the sphericity of maize seeds. The absence of significant differences in terms of sphericity indices, as shown in Table 2, suggests that the shape of a maize seed may rarely be affected by weed competition. However, other parameters such as bulk density, true density and porosity to weed competition should be evaluated to determine which traits can be affected by weed competition. This information is needed to design more accurate and productive agricultural machinery [55]. Our data agree with the results reported by other authors in other parts of the world. Soltani and colleagues report the effects of weeds on lowering the yield of corn crops in America and Canada [56]. Norsworthy and Oliveira identify the best time during the development of *Zea mais* to monitor weed growth [57]. Finally, other authors propose some strategies to be adopted to protect the yield of corn crops in the presence of weeds [58,59]. All these data confirm the need to better understand the biological systems involved in the interaction between the multiple species of weeds and crops of agronomic value.

## 5. Conclusions

The presence of weeds is the main constraint on crop production and even the adoption of more effective weed control measures cannot eliminate their negative effects. The susceptibility of cultivated plants to weeds can vary depending on the density and species of the weeds. *D. stramonium* and *X. Strumarium* are the most common and most studied weeds in maize crops. These weeds affect plant growth, seed production and biomass. However, the effects of weeds on the physical and engineering properties of maize seeds were still not known today. Indeed, the innovativeness of this work was to quantify the effects of *D. stramonium* and *X. Strumarium* on maize seed measurements such as total size, diameter, sphericity, surface area and seed volume. The use of these parameters finds considerable application in the evaluation of the healthiness of the plant and above all in post-harvest processing. Indeed, alterations in the physical and engineering properties of crops can cause reductions in machinery efficiency and product loss. In this work, we evaluated the effect of weed competition on the geometric characteristics of maize seeds. Maize seeds characteristics including length, width, breadth, aspect ratio and equivalent diameter were decreased by weed competition likely due to facing crop plants with severe limitation of growth requirements such as moisture, nutrients and light. Some properties were not consistently affected by weeds during of the two-year dataset, which suggest that evaluation of the other properties such as bulk density, true density and porosity is needed for determine weed competition effect on properties, which are important in design agricultural machinery. Overall, we have concluded that the negative effects on cleaning, grading and separation caused by the changes in the crop seeds grown in the presence of weeds should be considered in the design of agricultural machinery.

Despite the results collected, further studies need to be conducted to better define the mechanisms underlying the competition between weeds and corn crops. In particular, in future studies, all the metabolomic aspects that participate in the interaction in the soil and in the air between weeds and crops for agronomic purposes will have to be considered.

## Figures and Tables

**Table 1:** Analyses of variance of the effects of weed species (S), weed density (D) and their interaction on maize seed properties obtained in two-field experiment conducted in 2006 and 2007.

S.O.V	df	Length	Width	Thickness	Aspect Ratio	Surface Area	Volume	Sphericity	Equivalent Diameter	Angle of Repose	Seed Weight
						*p*-Value					
Year (Y)	1	0.0001	0.32	0.19	0.01	0.15	0.12	0.53	0.11	0.71	0.06
Block [Y]	4	0.60	0.08	0.12	0.91	0.01	0.00	0.13	0.00	0.40	0.27
Density (D)	4	0.00	0.00	0.12	0.02	0.01	0.00	0.42	0.00	0.01	0.00
Species (S)	1	0.49	0.35	0.44	0.40	0.87	0.81	0.24	0.85	0.13	0.32
D × S	4	0.22	0.52	0.80	0.16	0.92	0.89	0.14	0.91	0.22	0.44
Y × D	4	0.58	0.10	0.13	0.58	0.15	0.10	0.21	0.17	0.92	0.38
Y × S	1	0.18	0.35	0.46	0.12	0.33	0.34	0.37	0.33	0.83	0.09
S × D × Y	4	0.17	0.89	0.36	0.51	0.43	0.48	0.68	0.35	0.45	0.24
Error	36	-	-	-	-	-	-	-	-	-	-

**Table 2:** Basic geometric characteristics of maize seeds as affected with weed competition *.

Density	Length (mm)	Width (mm)	Thickness (mm)	Aspect Ratio	Equivalent Diameter (mm)
0	13.44 ^a^	8.33 ^a^	4.45 ^a^	61.83 ^b^	8.19 ^a^
4	12.16 ^b^	7.73 ^b^	3.84 ^a^	63.66 ^ab^	7.41 ^b^
8	11.47 ^bc^	7.50 ^b^	3.62 ^a^	65.50 ^ab^	7.07 ^b^
12	11.14 ^c^	7.59 ^b^	3.62 ^a^	68.33 ^a^	7.03 ^b^
16	10.8 ^c^	7.39 ^b^	3.57 ^a^	68.16 ^a^	6.9 ^b^

* Due to lack of significant weed species and year * treatment effects, data of 2006 and 2007 were pooled. Within the columns, mean values (*n* = 12; two years, two weed species and three replications) with the same superscript letters are not significantly different based on Tukey’s studentized range test (*p* ≤ 0.05).

## Data Availability

The data presented in this study are available on request from the corresponding author.
